# Determinants and timing of lifestyle changes in postmenopausal breast cancer survivors: A longitudinal qualitative study

**DOI:** 10.1371/journal.pone.0337089

**Published:** 2025-11-24

**Authors:** Sandra J. M. van Cappellen – van Maldegem, Anja de Kruif, Jacob C. Seidell, Lonneke V. van de Poll – Franse, Floortje Mols, Meeke Hoedjes

**Affiliations:** 1 Department of Medical and Clinical Psychology, Center of Research on Psychological disorders and Somatic diseases, Tilburg University, Tilburg, The Netherlands; 2 Department of Epidemiology and Biostatistics, Amsterdam Public Health (APH), VUmc, Amsterdam, The Netherlands; 3 Department of Nutrition, Dietetics and Lifestyle, School of Allied Health, HAN University of Applied Sciences, Nijmegen, The Netherlands; 4 Department of Health Sciences, VU University Amsterdam, Amsterdam Public Health (APH), Amsterdam, The Netherlands; 5 Department of Research and Development, Netherlands Comprehensive Cancer Organisation (IKNL), Utrecht, The Netherlands; 6 Division of Psychosocial Research and Epidemiology, The Netherlands Cancer Institute, Amsterdam, The Netherlands; Tsinghua University, CHINA

## Abstract

**Background:**

Adopting and maintaining favorable lifestyle changes can be challenging for postmenopausal breast cancer (PMBC) survivors. Understanding their experiences and needs can help tailor lifestyle support throughout their care journey.

**Purpose:**

This qualitative study explored 1) perceived determinants relevant for favorable lifestyle change among PMBC survivors, 2) the needs and preferences of PMBC survivors regarding lifestyle support, and 3) perceived determinants relevant for preferred timing of lifestyle support among PMBC survivors.

**Methods:**

A total of 42 in-depth longitudinal interviews were conducted at one year (n = 24) and 1.5 years (n = 21) following a breast cancer diagnosis. Data were analyzed using thematic analysis.

**Results:**

Our analysis revealed 12 main themes: 1) 3 themes describe determinants that may affect the process of favorable lifestyle change (type of lifestyle behavior, previous attempts to change lifestyle, comorbidities), 2) 3 themes express PMBC survivors’ needs for lifestyle support from health care professionals (HCPs) (need for information, need for activation by lifestyle support, need for effective communication by HCPs), 3) 6 themes describe determinants influencing PMBC survivors’ preferred timing of addressing these needs (type of treatment, coping style, previous experience with cancer, personality, motivation for lifestyle change, social support).

**Conclusions:**

Lifestyle support should consider psychological and physical effects of breast cancer diagnosis and treatment, which may hinder some PMBC survivors’ ability to make favorable lifestyle choices. Our results suggest that HCPs should offer ongoing individualized lifestyle support throughout the care continuum to meet the varied needs of PMBC survivors.

## Introduction

The development of postmenopausal breast cancer (PMBC) is, at least in part, associated with lifestyle factors (e.g., physical inactivity, alcohol consumption) and body fatness (e.g., adult weight gain, increased body fat) [1–7]. Compared to women without cancer, PMBC survivors also face an increased risk of second primary cancers (e.g., with a two- to fivefold increased risk for second primary breast cancers) [3], type II diabetes mellitus [4], cardiovascular disease [5], mortality [6], and a reduced health-related quality of life [8,9]. To mitigate these risks and improve quality of life [10–15], lifestyle and bodyweight recommendations have been issued by the World Cancer Research Fund [1,16].

**Fig 1:**
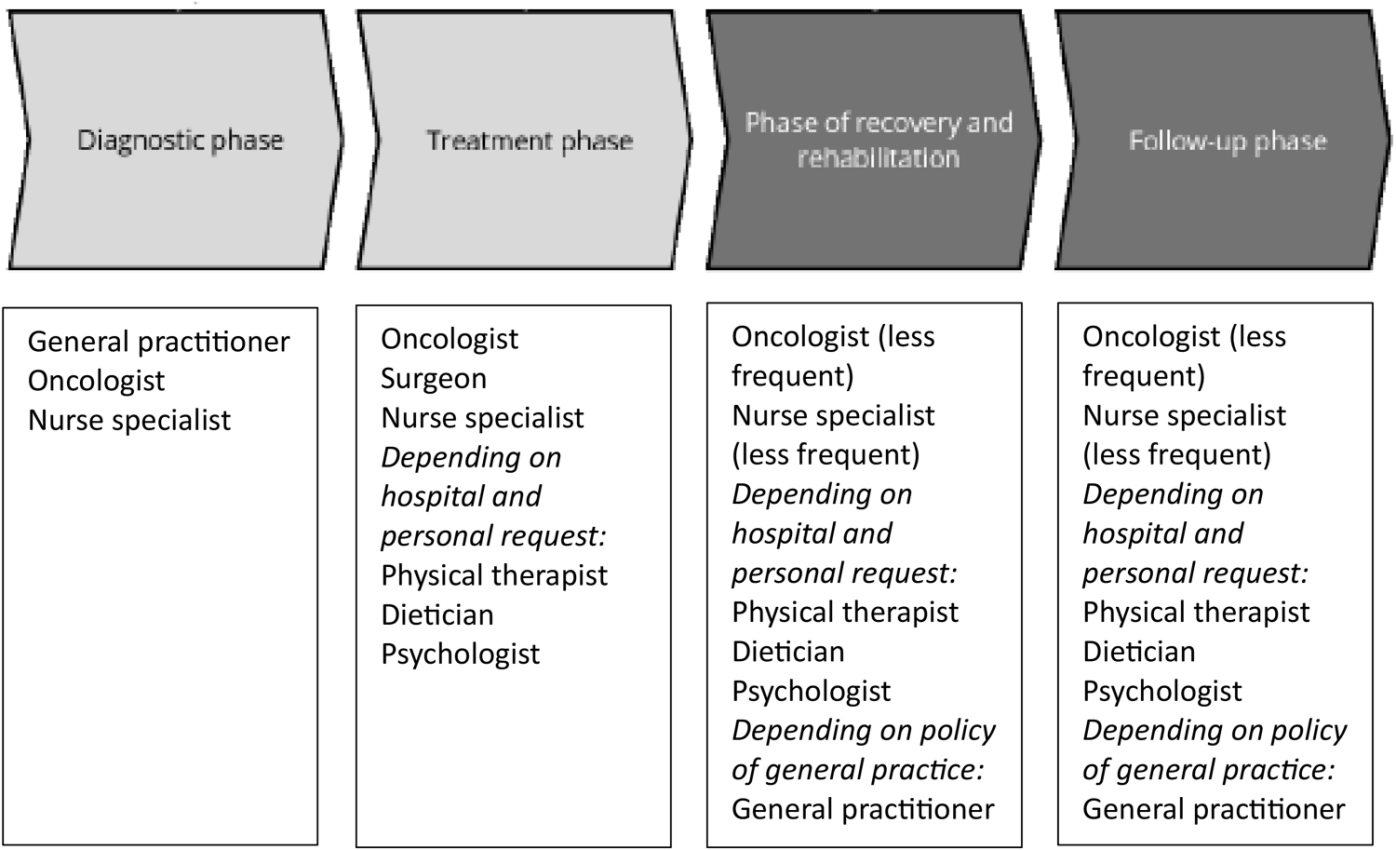
Overview of the patient journey of PMBC survivors, including the most common healthcare professionals involved at each stage.

Although a range of healthcare professionals (HCPs), including oncologists, nurse specialists, general practitioners, dietitians, physical therapists, and psychologists, could play a pivotal role in promoting these favorable lifestyle behaviors [17–19], this is yet not consistently integrated into clinical care [20]. After being diagnosed with breast cancer, many women undergo surgery, and additional therapy (e.g., chemotherapy, radiation therapy, hormonal therapy), followed by a period of follow-up care. During this trajectory, they encounter various HCPs (see Fig 1 for an overview of a typical PMBC survivors’ patient journey).

The transitions through the stages of active treatment to survivorship shown in this patient journey may be accompanied by changes in daily routines and lifestyle behaviors. Previous research indicates that indeed weight gain is common among PMBC survivors following diagnosis, driven by factors like chemotherapy, low levels of physical activity, and increased caloric intake [21]. Given the high prevalence of overweight and obesity in this group, along with the lack of structural integration of lifestyle counseling in clinical care, further research into effective strategies for promoting sustained adherence to lifestyle and body weight recommendations is essential.

For PMBC survivors, maintaining lifestyle changes can be more challenging than for the general population due to the physical, psychological, and social impacts of cancer and its treatment [22]. Favorable behavior change is not a single event [23]. Instead, it can be seen as a process through several stages of behavioral change (i.e., Transtheoretical Model of Change) [23–25]. The stages of change are: precontemplation (no intention to change), contemplation (awareness of the problem), preparation (planning to act), action (modifying behavior), and maintenance (consolidating changes and preventing relapse) [26]. Every stage is characterized by its own struggles. Lifestyle support can be tailored to an individuals’ needs by identifying their position in the change process. Offering lifestyle support that is not tailored to an individual’s stage of lifestyle change may lead to resistance to change [27].

Following favorable lifestyle changes, relapse into previous behaviors is common [27–29]. While there are many successful short-term lifestyle interventions for cancer survivors, research has shown that sustaining these favorable changes is challenging once the intervention program ends [30–32]. Understanding survivors’ readiness for lifestyle change and helping PMBC survivors to anticipate relapse can improve the change process [27].

In addition, attrition from lifestyle interventions is a frequent barrier to achieving successful lifestyle changes [33]. Specifically, attrition ranges from approximately 10% to 80% have previously been reported, depending on the setting and type of the intervention [34–37]. Those not completing an intervention often lack the support needed to develop skills for relapse prevention or maintenance strategies [38–40]. For this reason, previous studies have stressed the importance of tailoring interventions to the needs and preferences of intervention participants [33]. Moreover, a different approach for lifestyle support is needed for those with and without a perceived need for lifestyle support [41]. A personalized approach regarding the need for lifestyle support may increase success of interventions aimed to improve lifestyle and aimed at maintenance of lifestyle improvement [33,41]. However, there is limited understanding of the needs and preferences of PMBC survivors regarding lifestyle support that can help them complete lifestyle interventions and maintain long-term favorable lifestyle changes.

Throughout the breast cancer care continuum, a PMBC survivor’s readiness for change, need for support, and the effects of breast cancer and its treatment can change over time, influencing adherence to lifestyle and body weight recommendations. Thus, delivering a personalized lifestyle intervention requires considering the optimal timing for each individual survivor [42–44]. To date, studies on the optimal timing of lifestyle interventions for cancer survivors have yielded inconclusive results and did not specifically target PMBC survivors [20,42,43]. Additionally, these studies overlooked the individual determinants and characteristics of cancer survivors, which are pivotal for tailoring the most effective lifestyle interventions at key moments in the care trajectory. Tailoring lifestyle support in this way aligns with the principles of patient-centered care, which has been shown to improve outcomes across various clinical contexts [45].

Therefore, in this qualitative study, we aim to explore 1) perceived determinants relevant for obtaining and maintaining a favorable lifestyle among PMBC survivors, 2) the needs and preferences of PMBC survivors regarding lifestyle support, and 3) perceived determinants relevant for preferred timing of lifestyle support among PMBC survivors.

## Methods

### Study design

This study used a longitudinal interpretative qualitative design. The standards for reporting qualitative research (SRQR) were used [46]. This qualitative exploration was embedded in the OPTIMUM-study, a longitudinal observational study aiming to gain insight on the optimal timing and methods of lifestyle support in PMBC survivors. The OPTIMUM-study included measurements of 664 PMBC survivors at several time-points during their care trajectory (i.e., 4–6 months, 1 year, and 1.5 year following diagnosis breast cancer) [47]. Quantitative measurements included questionnaires, blood draws, accelerometer, and a food diary. Qualitative measurements entailed interviews, focus groups, and a Delphi-study.

In-depth interviews were held by the first author (SvC) who is an experienced qualitative researcher and second author (AdK) who is an experienced qualitative researcher in the field of oncology. Before the interviews, there was no personal involvement with the participants. The last author (MH) has ample experience with qualitative research and was available for consultation and content discussion during the interview and analytic phase.

### Participants and sampling

Patients were eligible for inclusion if they were diagnosed with breast cancer, were postmenopausal (i.e., not having menstruated for at least 1 year) at diagnosis, and were able to read and understand Dutch. Baseline questionnaires of the OPTIMUM-study were used to provide background information for purposive sampling. Purposive sampling was conducted based on variation in adherence to lifestyle and bodyweight recommendation (including BMI, and variation in weight loss/gain following diagnosis), readiness for favorable lifestyle change, and need for support to be able to improve lifestyle to obtain a representative sample of PMBC survivors. To illustrate the sociodemographic and clinical characteristics of the participating patients an overview is presented in Table 1. Based on these criteria, 30 PMBC survivors were invited for 2 longitudinal in-depth interviews at approximately 1 year and 1.5 year following diagnosis, of which 24 consented to participate. The first round of interviews took place from January 2020 till April 2020 (n = 24). The second round of interviews, 1.5 year following diagnosis, were executed from September 2020 till December 2020 (n = 21). At 1 year following diagnosis the interviews focused on PMBC survivors’ experiences, lifestyle and bodyweight prior to the diagnosis breast cancer, the diagnosis of breast cancer, the treatment, and in some cases early recovery from diagnosis and treatment. At 1,5 year following diagnosis the interviews mainly focused on PMBC survivors’ experiences following treatment, on recovering and rehabilitating, and on the time thereafter (See Fig 1). Participating PMBC survivors were treated at 7 hospitals in the Netherlands: Sint Jansdal (Harderwijk); Elisabeth TweeSteden Ziekenhuis (Tilburg); VieCuri Medical Centre (Venlo); Alexander Monro Ziekenhuis (Bilthoven); Amphia Ziekenhuis (Breda); Canisius Wilhelmina Ziekenhuis (Nijmegen); Reinier de Graaf Gasthuis (Delft).

**Table 1:** Socio-demographic and clinical characteristics.

	Mean (SD)
***N*** ^***1***^	24
**Age (years)**	62 (6.2)
**BMI**	
BMI before diagnosis	28.9 (5.6)
BMI interview 1	28.7 (5.5)
BMI interview 2	28.5 (2.1)
	**N (%)**
**Partner (yes)**	20 (83.3)
**Education level**	
Low	3 (12.5)
Medium	11 (45.8)
High	10 (41.7)
**Employment (yes)**	12 (50)
**Comorbidities**	
0	6 (25)
1	7 (29.2)
≥ 2	11 (45.8)
**Tumor Stage** ^**2**^	
**0**	2 (10)
I	10 (50)
II	7 (35)
III	1 (5)
IV	0 (0)
Missing	4
**Treatment** ^**2**^	
Surgery (yes)	3 (15)
Surgery (yes), Radiotherapy (yes)	4 (20)
Surgery (yes), Chemotherapy (yes), Radiotherapy (yes)	1 (5)
Surgery (yes), Chemotherapy (yes), Hormonal therapy (yes)	1 (5)
Surgery (yes), Chemotherapy (yes), Radiotherapy (yes), Hormonal therapy (yes)	5 (25)
Surgery (yes), Radiotherapy (yes), Hormonal therapy (yes)	6 (30)
Missing	4

^1^Note. 21 longitudinal interviews with postmenopausal breast cancer survivors were conducted at 1 year and 1.5 year following their diagnosis of breast cancer. For 3 postmenopausal breast cancer survivors there was 1 interview conducted at 1 year following diagnosis.

^2^Note. Clinical data (i.e., tumor stage and treatment) of 4 postmenopausal breast cancer survivors is unknown.

### Ethical considerations

The OPTIMUM-study was approved by the Medical Ethical Committee Brabant (NL66913.028.18). All participants provided written informed consent prior to participating in the OPTIMUM-study and provided oral approval for audio recording of the interviews.

### Data collection

Prior to the interviews, all participants completed a questionnaire to provide insight in demographic characteristics (i.e., age, education, employment, marital status), comorbidities, anthropometric factors (i.e., bodyweight and height), adherence to lifestyle recommendations (i.e., diet (Dutch Healthy Diet-Index [48]), physical activity (Physical Activity Scale for the Elderly [49]), smoking, alcohol, sleep (Pittsburgh Sleep Quality Index [50]), stage of readiness for lifestyle change (not ready: pre-contemplation/ contemplation; ready: preparation/ action/ maintenance; relapse [23]), and need for support (yes/no) for each lifestyle and bodyweight recommendation.

Data collection for the first round of interviews at 1-year post-diagnosis occurred in March and April 2020. The second round of interviews at 1.5-year post-diagnosis were planned August and September 2020. The interviews at 1-year post-diagnosis were held at the home of the PMBC survivor before the onset of COVID-19 in March 2020 (n = 14). After the onset of COVID-19, the interviews were held by phone call or online videocall (n = 10). The interviews at 1.5-year post-diagnosis were held at the home of the PMBC survivor. It is not expected that the results of the phone call or online videocall interviews differ from the face-to-face interviews [51]. Prior to the interviews at home, additional time was spent entering the house, having a drink, and engaging in small talk. We believe the absence of these steps did not affect the PMBC survivors’ comfort in discussing personal issues, as the average duration and richness of data were comparable between home and phone/video interviews. A topic list was used to guide the interviews (see S1 File). After approximately every five interviews, MH, AdK, and SvC discussed the main findings in relation to the topic list to assess whether new information had emerged and to determine the appropriate point to conclude interviewing based on data saturationOn average, the interviews lasted 1 to 1.5 hours. All interviews were digitally audio-recorded and transcribed verbatim. To enhance trustworthiness of the study, following each interview a summary was made and checked for correctness by the PMBC survivor (member checking).[52]

### Data analysis

Thematic analysis, based on the constructivist research paradigm, was performed using Atlas.ti version 22 [53]. The thematic analysis was conducted in six iterative phases [53]:1) familiarizing with the data reading the transcripts; 2) independently open coding of all transcripts by 4 research assistants (RAs) and 1 researcher (SvC), all trained in using Atlas.ti. First, ten transcripts were independently and inductively coded by SvC and 4 RAs. In addition to the standard six phases, an intermediate step creating a structured code scheme was integrated at this time point [54]. To create the code scheme, initial codes were discussed during weekly meetings by SvC and 4 RAs. Following, once the code scheme was defined, the allocated codes in the first 10 transcripts were deleted and all interviews were (re)coded using the code scheme. New codes which were added to the code scheme were discussed to guarantee consensus; 3) categorizing the codes into potential themes. Two levels of themes were determined: main themes and subthemes. The themes and subthemes were discussed in group meetings between 4 RAs and 1 researcher (SvC), and between two researchers (MH and SvC) until consensus was reached; 4) review and refinement of the themes and subthemes (MH and SvC); 5) defining and refining themes by identifying a theme’s and subtheme’s main content (MH and SvC); 6) relating the results to the research questions by identifying determinants relevant for adherence to lifestyle and bodyweight recommendations, readiness for change, and need for support (MH and SvC).

## Results

**Fig 2:**
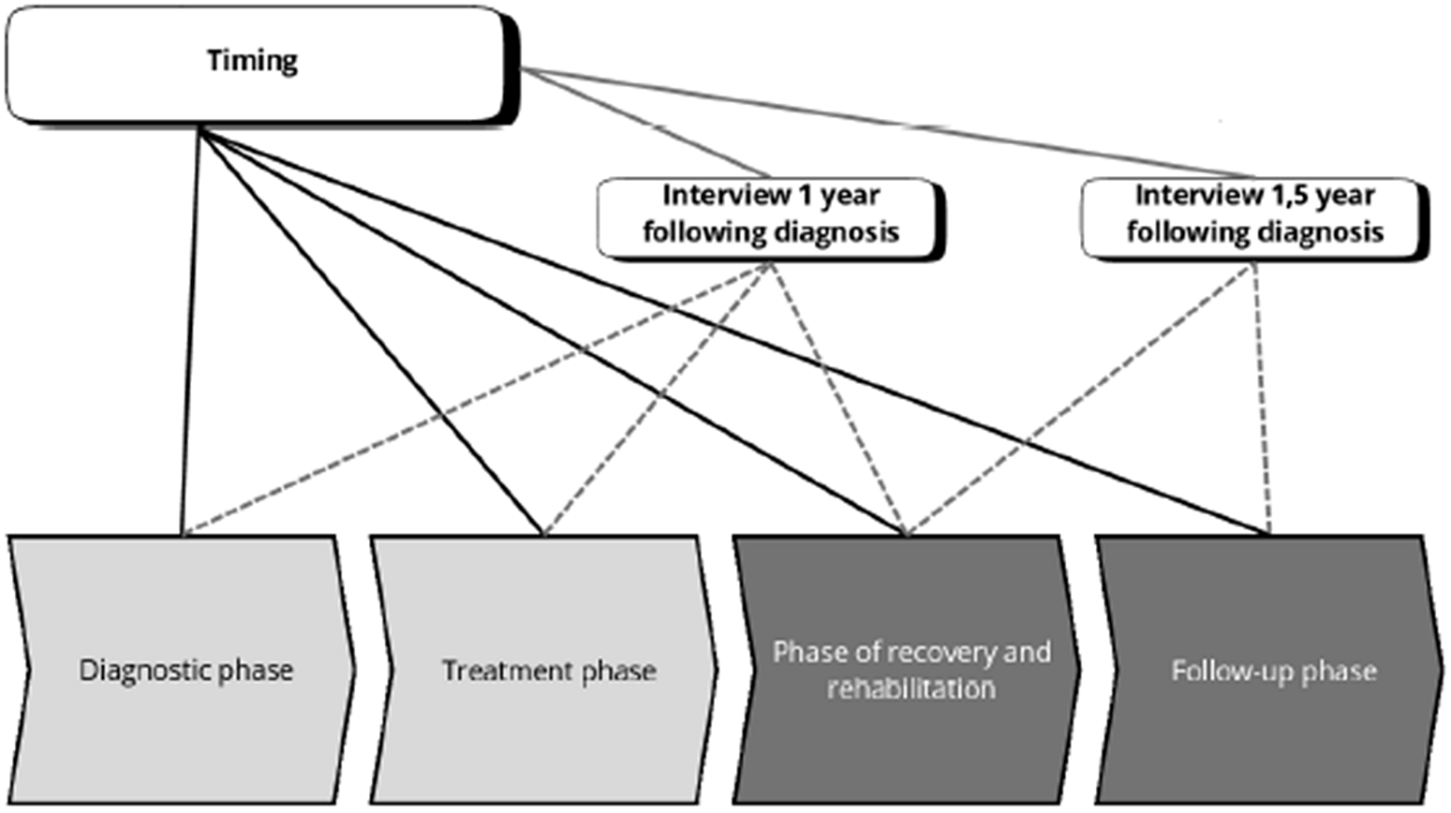
Visualization of the focus of the interviews at 1 and 1.5 years following diagnosis.

Twenty-one interviews were conducted both at 1 year and 1.5 year following diagnosis. Fig 2 illustrates the focus of the interviews conducted at 1 and 1.5 years following diagnosis, alongside the potential timing of professional lifestyle support.

For three PMBC survivors, only an interview at 1-year post-diagnosis was conducted. Specifically, one PMBC survivor felt too confused by the postponed medical results due to COVID-19, another no longer wished to participate as she did not want to be reminded of her breast cancer diagnosis and treatment, and the third did not specify a reason for withdrawing. Characteristics of the study population (n = 24) are presented in Table 1. Our analysis revealed twelve main themes. The results begin with discussing determinants that may affect the process of favorable lifestyle change (aim 1; 3 themes), followed by the needs expressed by PMBC survivors for lifestyle support from HCPs (aim 2; 3 themes). Finally, we will describe determinants influencing the preferred timing of PMBC survivors in addressing these needs (aim 3; 6 themes). **Main themes** will be printed in bold and *sub-themes* in italics.

### Aim 1: Determinants affecting the process of favorable lifestyle change

#### Type of lifestyle behavior.

**Fig 3:**
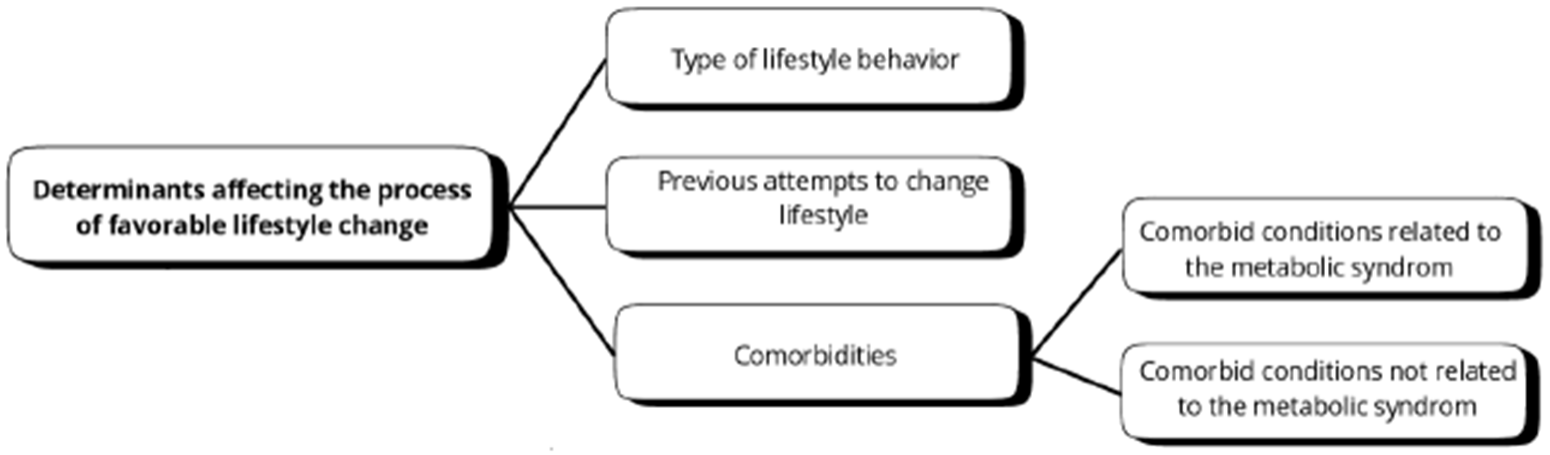
Overview of themes and subthemes for determinants affecting the process of favorable lifestyle change.

Awareness and knowledge of potential favorable lifestyle changes appeared to vary depending on the type of behavior (Fig 3). Most PMBC survivors reported focusing on weight loss when discussing favorable lifestyle changes. As a result, the most mentioned lifestyle changes involved adjusting their diet and increasing physical activity. In general, PMBC survivors seemed unaware of the potential to make lifestyle changes related to sleep. While some survivors mentioned experiencing sleep problems, few reported seeking medical advice or help for their sleep issues. In contrast to lifestyle changes related to diet and physical activity, which were described as more gradual, quitting alcohol and smoking seemed to happen more abruptly. A minority of PMBC survivors who consumed high levels of alcohol, as well as those who smoked, mentioned they quit prior to the onset of treatment for health benefit. *“After diagnosis, I heard alcohol could cause cancer. During chemotherapy, I wanted to be as healthy as possible, so I immediately stopped drinking”.* Some others reduced their alcohol consumption or quit smoking at the start of treatment because it didn’t align well with the treatment.

#### Previous attempts to change lifestyle.

Some PMBC survivors shared lifelong concerns about their weight and lifestyle, coupled with high health awareness and knowledge about making lifestyle changes (Fig 3). However, they also mentioned frequently struggling with relapse and maintaining these changes over time. *“I was* *always searching for miracle ways to lose weight. I tried many diets and could manage when I felt good, but I always fell back into my old habits”.* Others had no prior experience with lifestyle changes, feeling it was unnecessary due to a healthy weight or a lack of health issues (Fig 3). *“I always weigh around 55 kilograms. If my weight increases slightly, I immediately pay attention to ensure to bring it back down to 55 kilograms again”.* Additionally, some had made some recent lifestyle changes due to a recent diagnosis (e.g., diabetes type 2, need for hip replacement), aging, or a relative’s health issues (e.g., cancer diagnosis of partner).

#### Comorbidities.

The presence of comorbid conditions was reported to affect adherence to lifestyle and bodyweight recommendations and lifestyle change (Fig 3). In general, PMBC survivors suffering from *comorbid conditions related to the metabolic syndrome* (e.g., type 2 diabetes, high blood pressure, etc.) mentioned to have received lifestyle support prior to their breast cancer diagnosis. However, as they perceived making favorable lifestyle changes to be hard, not all of them succeeded in making favorable changes following lifestyle support. They were, however, often aware of the potential health benefits of making favorable lifestyle changes in relation to their comorbid condition. *“I changed my diet when I was diagnosed with type 2 diabetes. I started eating less carbs and started to swim”.* PMBC survivors suffering from c*omorbid conditions not related to the metabolic syndrome* (e.g., hip replacement, knee injury, etc.) less often received lifestyle supportprior to the diagnosis of breast cancer and may benefit from more basic lifestyle support and guidance. *“I’m usually active, but with my hip issues, I can’t exercise. I tried strength training, but developed edema, and was told it wasn’t suitable after breast cancer surgery.”*

### Aim 2: PMBC survivors’ needs regarding lifestyle support by HCPs

#### Need for information.

Most PMBC survivors told they would have preferred to receive more information regarding the *relationship between lifestyle and cancer*. There were a lot of PMBC survivors mentioning to be unaware of the relation between lifestyle and cancer. Looking back in retrospect, they would have preferred to receive this information. “*After diagnosis, I heard that alcohol could cause cancer, I didn’t know that”.* PMBC survivors also mentioned a need for *information with respect to nearby lifestyle services*, for example by means of a list for referral. This list could, for example, include physical therapists with experience in oncology and information about local rehabilitation programs available in the area. They also mentioned to prefer to receive *practical information to make favorable lifestyle changes*, for instance, a suggested weekly diet with sample meals or a specific exercise program. *“I would like to have a nutrition plan, specifically for cancer survivors”.*

#### Need for activation by lifestyle support.

PMBC survivors in rehabilitation programs with supervised physical activity reported enjoying the exercise and experienced more fitness and accompanying health benefit. They mentioned “being activated” and continued it independently afterwards. However, PMBC survivors felt less activated with diet consultations, finding it difficult to incorporate dietary advice into their daily lives without prior active guidance and behavioral practice. *“I asked the dietitian how to maintain healthy liver values. She told me to follow the ‘Dutch food-based dietary guidelines* [55], *but I did not get practical advice, like which meals to prepare and”.*

#### Need for effective communication by HCPs.

PMBC survivors’ progress through the stages of change was influenced by lifestyle advice from health care professionals. For example, PMBC survivors in the precontemplation stage (i.e., not yet considering change or being unwilling or unable to change), mentioned that discussing lifestyle with HCPs in relation to their treatment and prognosis made them start to consider changing behavior. Although not all PMBC survivors received lifestyle advice, those who did found it often motivating, particularly when they saw their healthcare provider as credible and supportive. *“I don’t remember exactly how it was phrased, but it did make me think, “Oh, I should eat a lot of dairy products, okay!”.*

However, if communication lacked empathy, survivors were less likely to consider the advice seriously, emphasizing the importance of effective communication in encouraging lifestyle changes. *“My oncologist said as a starter: ‘Oh you are really fat.’ That was not nice, since then I did not feel well when meeting the oncologist”.*

### Aim 3: Determinants influencing the preferred timing for lifestyle support among PMBC survivors

**Fig 4:**
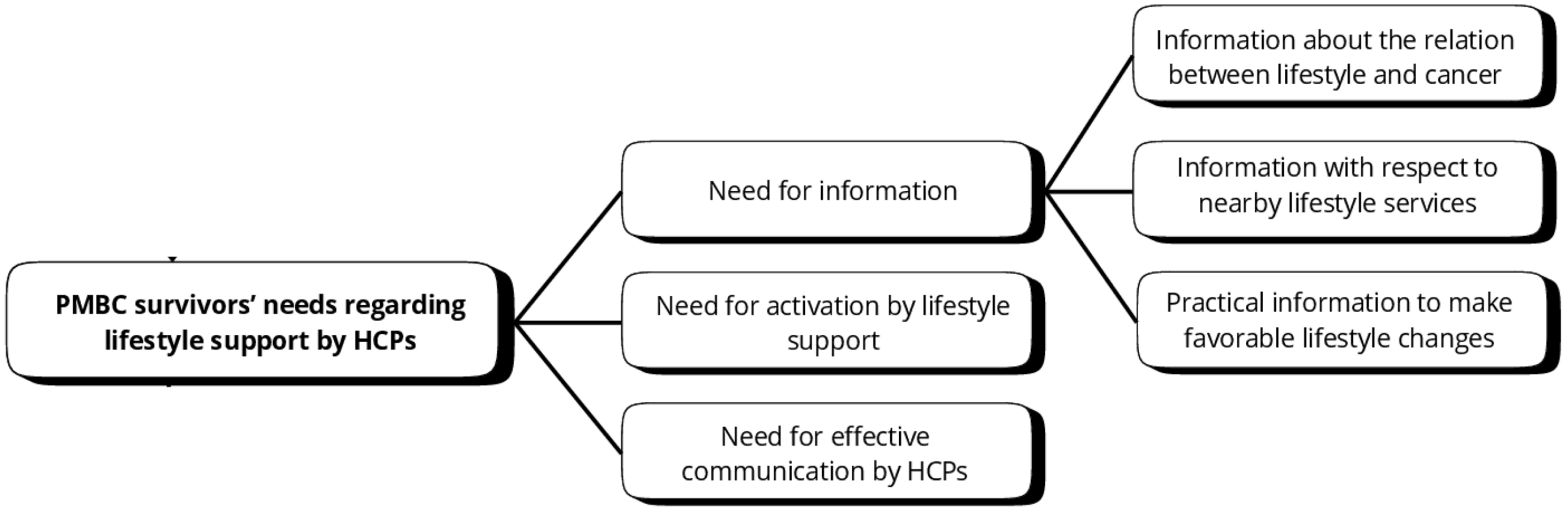
Postmenopausal breast cancer survivors’ needs regarding lifestyle support by health care professionals.

**Fig 5:**
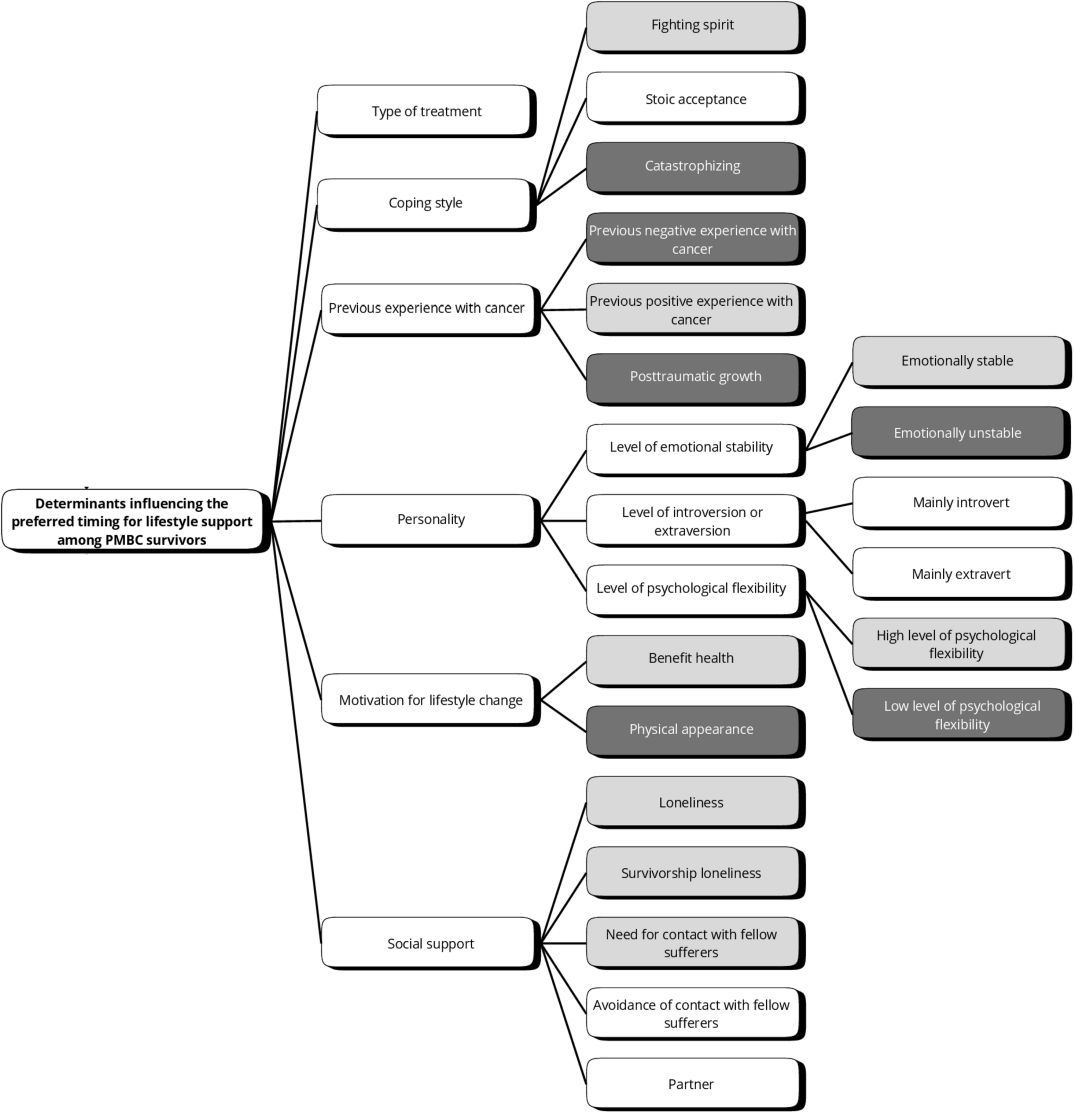
Overview of themes and subthemes and association with preferred timing for lifestyle support by PMBC survivors. Note. Subthemes in light grey correspond to a preferred timing of lifestyle support early in the care continuum (diagnostic phase and treatment phase). Subthemes in dark grey correspond to a preferred timing of lifestyle support later in the care continuum (phase of recovery and rehabilitation and follow-up phase).

The PMBC survivors’ preferred timing of lifestyle support may be influenced by the following determinants (6 themes) (See Fig 4). Specifically, Subthemes in light grey (Fig 5) correspond to a preferred timing of lifestyle support early in the care continuum (diagnostic phase and treatment phase). Subthemes in dark grey correspond to a preferred timing of lifestyle support later in the care continuum (phase of recovery and rehabilitation and follow-up phase). The colors (light grey and dark grey) are aligned with the phases of the care trajectory shown in Fig 2.

#### Type of treatment.

The lifestyle support needs of PMBC survivors who received chemotherapy differed from those of survivors who did not undergo chemotherapy. Those receiving chemotherapy mentioned a lot more practical lifestyle-related questions, for example questions concerning diet (e.g., in relation to vomiting, taste and smell changes, etc.) and exercising. While receiving chemotherapy, a lot of survivors mentioned experiencing overload, they felt to have started a rollercoaster in which they just go along. These PMBC survivors mentioned they had no capacity left to incorporate lifestyle changes at this time point. For example: *“During chemotherapy you are busy with only one thing: surviving”.* Both groups reported similar issues with respect to sleep, especially in relation to cancer-related fatigue.

#### Coping style.

Based on the experiences women shared during the interviews, it became apparent that the ability to make potential favorable lifestyle changes following breast cancer diagnosis and treatment seemed to be related to coping style. Three types of coping styles came forward during the interviews. First, some PMBC survivors mentioned experiencing ‘*fighting spirit*’ following diagnosis. They mentioned an optimistic attitude, accompanied by a search for information regarding breast cancer (treatment) and everything in their power to survive the cancer. For most of these PMBC survivors, favorable lifestyle change was a way to feel a sense of control. *“Then I thought, you have to stay positive and tell yourself, ‘I’m going to fight this battle, and when I can’t fight anymore, that’s when it ends.’ I also thought, “If you [oncologist and surgeon] handle what I can’t control, and I do everything I can, then I’ll know I’ve done all I could”.* These PMBC survivors expressed a preference for receiving lifestyle support as early as possible in the care continuum. This would provide them with reliable information and save them the time spent searching for lifestyle and cancer-related guidance.

Other PMBC survivors showed ‘*stoic acceptance*’ following diagnosis. These PMBC survivors did their best to ignore the breast cancer and any treatment-related symptoms by focusing on other positive aspects of life. They sought to accept the breast cancer diagnosis and, in turn, continue living as normally as possible.

“*That illness has actually had so little impact on me.*”

*“I thought as long as I don’t get any aftercare everything is fine….I actually never think about it”.* These PMBC survivors did not express a preference regarding timing for lifestyle support.

Third, some PMBC survivors mentioned ‘catastrophizing’ following the (suspicion of) their diagnosis of breast cancer. They described feeling helpless and hopeless, often experiencing excessive worry about the future and their prognosis, accompanied by high levels of stress and anxiety. A PMBC survivor mentioned: *“Then a scan is done, and I am always happy when nothing shows up. Because I still always have cancer worry. Because I still always have the feeling that something isn’t right.”.* Another PMBC survivor said: *“Your trust in your body is really gone, especially in the beginning. It slowly improved, but now it’s resurfacing with an upcoming check-up. This morning, I checked myself and thought. ‘What is that weird feeling? Could it be back*??’*”.* PMBC survivors with a tendency to catastrophize mentioned being completely focused on the breast cancer diagnosis and its treatment at the beginning of their care journey, leaving no room for lifestyle advice or change. Later in the care continuum would be the preferred time for lifestyle support for these survivors.

#### Previous experience with cancer.

From women’s experiences it was derived, that a previous experience with cancer of either themselves or someone close to them, may affect the current experience with the cancer diagnosis and its treatment. If someone close struggled a lot during treatment or died, the cancer diagnosis was perceived as more negative and fearful. This *negative previous experience with cancer* may affect the choices made following diagnosis and needs regarding (lifestyle) counselling compared to PMBC survivors with a previous positive experience. PMBC survivors with such a previous negative experience expressed a high need for control, a high need for information, high levels of fear and lower belief in health care professionals compared to PMBC survivors with a previous positive experience. *“Deciding on radiation was the hardest decision of my life. I did not know the right choice. My husband had 85 rounds before he passed and became very sick, but I wanted certainty for my daughters and radiation would give me 15% higher chance of survival”.* PMBC survivors with negative experiences mentioned to be very focused on the treatment, with high levels of stress, therefore unable to focus on their lifestyle early during the care continuum.

Others mentioned to have a *previous positive experience with cancer*. For example, they mentioned that they knew someone who survived cancer without many (additional) medical problems and good contact with the health care professional, or they reported to have previously survived cancer without complications themselves. Also, they mentioned higher levels of trust in health care professionals. These PMBC survivors preferred lifestyle support early during their care continuum.

Some PMBC survivors seemed to have experienced *posttraumatic growth* following their diagnosis of cancer. They reported an increased appreciation of life and feelings of strength, which caused them to make successful favorable (lifestyle) changes. *“In October, I started asking myself: who am I, what do I want, what do I want from my work and my life? I was told that losing weight* *could improve my chances of survival, so I changed my diet and began walking. I ended up losing 12 kg.”.* For PMBC survivors experiencing posttraumatic growth, lifestyle support early during the care continuum seems beneficial.

#### Personality.

The experiences women reported suggest that personality characteristics seem to affect the way in which PMBC survivors deal with the diagnosis and favorable lifestyle change. PMBC survivors who seemed *emotionally stable* mentioned to plan anything in their power to survive cancer and enjoy life as much as possible, in contrast to the PMBC survivors who considered themselves to be more *emotionally unstable*. Emotionally stable PMBC survivors would prefer receiving lifestyle advice early in the care continuum to implement favorable changes that could improve their prognosis. The more emotionally unstable PMBC survivors needed all their energy to go through what they called “the rollercoaster” following the diagnosis, which to them included hospital visits, treatments, and psychological uncertainty. These PMBC survivors benefit from lifestyle support later during the care continuum, for example during recovery and the follow-up phase. An emotionally unstable PMBC survivor mentioned: *“My glass is always ‘half empty’. Right now, I don’t feel balanced. At first, I pretended to be stronger and said everything was fine, but that’s no longer the case. Now, I’m getting help from a psychologist and talk about how I really feel.”.*

In addition, PMBC survivors seemed to differ in the extent to which they dared to ask for help, including lifestyle advice or support. According to some PMBC survivors, this may be related to their personality, to their *level of introversion or extraversion*, with the latter generally feeling freer to ask for support and advice. An extravert PMBC survivor mentioned: *“I just asked for a referral, and I got it”.* And another extravert PMBC survivor: *“I have noticed that many people I* *meet during chemo or at the gym often complain about the hospital or say thinks are unclear, like ‘How am I supposed to know that? I always tell them: ‘If something is unclear, you can always ask again’”.* No preference regarding timing of lifestyle support was expressed based on level of introversion or extraversion.

Last, also the *level of psychological flexibility* of the PMBC survivor appeared to affect the way they dealt with the diagnosis and treatment. PMBC survivors who mentioned they normally are flexible, and ‘go with the flow’, seemed to be less affected by the breast cancer diagnosis and treatment and less sensitive to stress. For example: *“I am the type that always waits to see how things turn out and then see how it goes*”. These PMBC survivors prefer lifestyle support early during the care continuum, in contrast to PMBC survivors with lower psychological flexibility, who preferred lifestyle support later during the care continuum.

#### Motivation for favorable lifestyle change.

The preferred moment of lifestyle support also depended on the PMBC survivors’ type of motivation they had to change their lifestyle. Some PMBC survivors mentioned they made favorable lifestyle changes to *benefit their health*. The diagnosis breast cancer or a different diagnosis in the past (such as type 2 diabetes) triggered them to start making favorable lifestyle changes. For example, a PMBC survivor mentioned: *“I changed my diet and worked on getting fitter to improve my chances of survival and for the health benefits”.* For these PMBC survivors, receiving lifestyle support at the time of diagnosis or shortly after could be beneficial. In addition, getting older and having family and peers with physical complaints were also mentioned as triggers to start making favorable lifestyle changes.

Other PMBC survivors mentioned that they primarily made favorable lifestyle changes to lose weight, focusing on their *physical appearance*. They appreciated looking slimmer and being able to fit into smaller-sized clothes. A PMBC survivor mentioned: *“I don’t find my body very attractive, which is why I want to lose weight”*. The PMBC survivors who focused on their physical appearance typically mentioned this later in their care journey. For them, receiving lifestyle support during the recovery phase or follow-up phase would be most beneficial.

#### Social support.

Various types of social support were mentioned during the interviews, including practical support (e.g., getting a ride to the hospital, receiving cooked meals), emotional support (e.g., having the opportunity to express what’s going on), and informational support (e.g., receiving information about treatment or lifestyle changes). PMBC survivors who expressed feelings of *loneliness* may require additional support compared to those with a larger social network. Loneliness was reported to affect self-care including healthy lifestyle behaviors. For example, a lonely PMBC survivor mentioned being unable to prepare a healthy dinner for herself at days receiving radiotherapy as she was just too exhausted, resulting in not eating at all on treatment days. She said: *“I started neglecting my self-care. I stopped cooking healthy meals for myself and would just have a slice of bread instead. I simply didn’t have the energy anymore”.* Lonely PMBC survivors may benefit from lifestyle support in a group setting early in the care continuum, as it could help connect them with others who are going through similar experiences.

Other PMBC survivors mentioned to be part of a social network, however, to still feel alone with respect to cancer. They experienced *survivorship loneliness* and wished to get in touch with fellow PMBC survivors to share experiences. A PMBC survivor said: “*I felt a lack of understanding from others. I didn’t look or feel sick, but in reality, I was seriously ill which was very difficult to deal with*”. This desire to share experiences included the wish to exchange both successful and unsuccessful lifestyle changes (e.g., tasty recipes during chemotherapy). PMBC survivors experiencing survivorship loneliness preferred lifestyle support in group setting early in the care continuum to get in touch with fellow sufferers.

Most PMBC survivors expressed a *need for contact with fellow sufferers* for emotional support and practical advice. In physical therapy groups, they felt more at ease with their bodies around other cancer survivors, as everyone was understanding. For PMBC survivors with a need for contact with fellow sufferers offering (group-based) lifestyle support early in the care continuum would be beneficial. In contrast, some PMBC *survivors reported avoiding contact with fellow sufferers*, fearing it would be emotionally overwhelming. They believed such interactions would lead to sadness and complaints, and this led some to reject rehabilitation invitations. A PMBC survivor avoiding contact with fellow sufferers mentioned: *“I didn’t want to go there. I thought they would tell all about bad experiences. That thought made me very anxious”.* These PMBC survivors would prefer to receive lifestyle support individually.

Many of the interviewed PMBC survivors had a *partner*. For part of them this was very positive, as they received emotional and practical support from their partner. *“At the hospital they advised me to exercise as much as possible during chemotherapy. So, my husband took me for a walk every day for at least 30 minutes”.* Some survivors felt hindered by partners who didn’t support favorable lifestyle changes, such as expecting the same meals or snacks. This lack of support made them feel alone in their efforts to fight cancer in every possible way.

## Discussion

In this longitudinal qualitative study, we found that relevant determinants of favorable lifestyle change in PMBC survivors included the type of lifestyle behavior being changed, previous experiences with lifestyle change, and the presence and type of comorbidities. In addition, we found that nearly all PMBC survivors expressed a need for information and support regarding their lifestyle and body weight in relation to cancer (survivorship). Additionally, PMBC survivors expressed a need for activation by lifestyle support and a need for effective communication by HCPs while providing lifestyle support. Moreover, the timing of this support appears to be influenced type of treatment, coping style, prior cancer experiences, personality traits, motivation for lifestyle change, and the availability of social support.

To the best of our knowledge, no existing studies on this topic have specifically focused on PMBC survivors. Therefore, we compared our findings with prior research conducted on breast cancer survivors as a whole or on the broader cancer population. Our findings align with previous studies signaling that lifestyle support is not structurally embedded in clinical care of PMBC survivors [17–20,56,57]. PMBC survivors expressed a need to receive information from their treating HCPs regarding lifestyle advice in relation to cancer. PMBC survivors did not mention a preferred HCP to provide this information. Moreover, in line with previous research, PMBC survivors see their treating HCPs as default experts and credible source to provide this information [58]. Important in this respect, is the alignment of a HCPs’ and PMBC survivors’ beliefs concerning an appropriate communication style [58,59]. If these do not align, we also noticed in our study, PMBC survivors value and trust their HCP less than PMBC survivors who are satisfied with their HCP [58,59]. As a result of this ineffective communication style (e.g., communication lacking empathy), PMBC survivors mentioned they were less likely to adhere to lifestyle and bodyweight suggestions made by their HCP.

In addition, similar to previous studies, PMBC survivors in the current study mentioned beneficial effects of an exercise-based revalidation program during treatment or thereafter such as feelings of fitness, distraction, and social contact with fellow sufferers [60,61]. In contrast, while receiving dietary consultation PMBC survivors missed demonstration of the behavior, behavioral practice and rehearsal, and contact with fellow sufferers. They reported that receiving dietary advice on paper or through explanation alone did not activate them. This finding is supported by a previous systematic review on the effectiveness of dietary interventions, which found that active dietary consultation, such as cooking demonstrations, teaching real-life skills like vegetable preparation, and hands-on cooking experiences, enhanced healthy eating habits and self-efficacy regarding favorable dietary choices [62,63].

The interviews revealed a global division of two groups of PMBC survivors over time. One group of PMBC survivors felt highly stressed and anxious, had a previous negative experience with cancer, felt powerless, and felt emotionally unstable after diagnosis, lacking the psychological flexibility to cope, and showed little need for lifestyle support early in the care continuum. Their need for lifestyle support emerged later, during the phase of recovery and rehabilitation or follow-up. In contrast, the other group of PMBC survivors showed a fighting spirit, had a previous positive experience with cancer, felt emotionally stable, and felt adaptable preferred lifestyle support early on, often during the diagnostic phase, or slightly later during treatment. Providing lifestyle support early saves the latter group time searching for reliable information and may improve its effectiveness. This finding can be explained by the theory of the Transtheoretical Model of Change [23]. Even though others may influence your motivational readiness for change, the decisional balance must shift in favor to new behaviors within the individual attempting change [23,64–66]. Increased levels of stress and anxiety, as well as physical complaints and treatment side effects, may hinder the internal decisional balance in favor of a healthier lifestyle at that time [64]. For this reason, it is important to ask PMBC survivors regularly during the care continuum if they would prefer to receive lifestyle support at that moment.

In addition, the social network of the PMBC survivor played a vital role in their ability to obtain or maintain a favorable lifestyle. Those who reported to receive social support could discuss their emotional wellbeing, weigh the pros and cons of a favorable lifestyle, and often exercised or shared healthy meals with family or friends. In contrast, survivors lacking social support, for example due to smaller social networks, felt the burden of self-care, especially during challenging times like chemotherapy or radiation, with some unable to prepare meals and resorting to simple options like a slice of bread for dinner. This is in line with previous research stating that partners [67,68], children [69,70], family [71,72], friends [73] and community members [74] can play an important role offering social support during periods of poor health and high stress. In particular, social support has been found to potentially mediate the effect of disease on reported wellbeing [75]. In line with this, PMBC survivors experiencing loneliness or survivorship loneliness, and those in need for contact with fellow sufferers, expressed a preference for group-based lifestyle support early in the care continuum.

### Strengths, limitations, and recommendations for future research

The qualitative design of the study enabled the collection of a complete overview of relevant determinants, without the risk of missing determinants caused by using a quantitative design with pre-selection of determinants. Another strength was the use of purposive sampling which led to a high variety of characteristics of participating PMBC survivors (i.e., variation in adherence to lifestyle and bodyweight recommendations, variation in readiness for favorable lifestyle change, and variation in need for support). In addition, techniques to enhance trustworthiness such as member checking and discussion among researchers during all phases of the qualitative analysis, including discussion of the determinants until consensus was reached, were performed [52]. As relevant determinants may change over time during the care trajectory, the longitudinal design of this study is also a strength.

Even though we used purposive sampling, it should be considered that most of our study sample consisted of moderate or higher educated PMBC survivors. This may limit the generalizability of the results since women with a lower level of education may experience additional or different determinants that are relevant to lifestyle change and support. For example, previous research has highlighted the potential influence of educational level on food choices. A lower education level may be associated with limited nutritional knowledge and low awareness of food-related issues, which can hinder adherence to lifestyle recommendations for a healthy diet [76]. Future studies may focus on PMBC survivors with a lower level of education to explore their needs and experiences regarding lifestyle support following their diagnosis. In addition, it should be considered that this study is executed in the Netherlands, where we have a solidarity-based healthcare system where everyone is required to pay for health insurance and thereby receives health care. For this reason, all PMBC survivors in het Netherlands receive the same care irrespective of their background. While financial incentives are less likely to influence preferences and experiences, other social economic or ethnicity related factors may still have been of influence in the experience of diagnosis of breast cancer and the subsequent treatment during our study. Future studies may also focus on PMBC survivors in countries with limited access to healthcare services to explore their experiences regarding lifestyle (support) and their cancer diagnosis and treatment. For example, educated and/or more affluent women may be more likely to seek medical or lifestyle care proactively whereas less affluent PMBC survivors may be less informed about treatment or lifestyle care options. Previous studies described a potential role of insurance status, income, socioeconomic status (SES), and ethnicity in this respect [77]. Specifically, previous studies indicate that minority and low-SES patients may receive care from different providers due to financial barriers, geographic factors, or insurance status. Variations in quality of care between providers may, in turn, contribute to socioeconomic and racial disparities in access to appropriate systemic therapies [78,79].

Due to the onset of COVID-19 we switched from interviews at the PMBC survivors home address to online interviews via Teams or by telephone to limit face-to-face social contact. This different setting may have influenced the richness of the data. However, based on previous studies comparing online and face-to-face interviews, we expect any effect to be minimal or negligible [51,80].

During this study, we mainly focused on experiences and preferences of PMBC survivors regarding professional lifestyle support. Due to this reason, we did not specify and highlight the potential distinct roles different HCPs can play. A valuable direction for future research would be to make this distinction and to further explore the potentially different roles that various health care professionals may fulfil.

### Implications for practice

Sleep problems and insomnia are common among cancer survivors [81,82]. However, we found that many PMBC survivors were unaware of available therapies or interventions for these issues, as only a few sought help from healthcare professionals. Increased awareness and attention to treatments for sleep problems by means of discussing sleep quality during consultation, increasing awareness of the importance of healthy sleep habits in waiting rooms (e.g., pamphlets or on screens), and offering sleep counselling, could benefit cancer survivors.

In addition, some PMBC survivors expressed a desire to connect with others, while others preferred to avoid it. Since lifestyle support, including rehabilitation, is often group-based, this difference should be considered in lifestyle care. A practical solution could be a buddy system, allowing individuals to connect with fellow survivors. Additionally, offering a social moment (e.g., drinking coffee or tea) after lifestyle sessions would accommodate both needs, those wanting interaction can stay, while others can leave.

In conclusion, we identified several determinants relevant to obtaining and maintaining a favorable lifestyle following the diagnosis of PMBC. The type of lifestyle behavior to be changed, earlier experience with lifestyle change, and the presence and type of comorbidities, were found to affect the process of favorable lifestyle change in PMBC survivors. Our findings suggest that nearly all PMBC survivors express a need for information regarding the association between lifestyle, bodyweight and cancer. In addition, some PMBC survivors wish to receive real-life active lifestyle support, including for dietary consultation. During provision of lifestyle support, it is important for HCPs to use an appropriate and empathetic communication style. The timing to meet these needs for lifestyle support depends on the PMBC survivors’ type of treatment she receives, their coping style, previous experience with cancer, personality, motivation for lifestyle change, and their social support system. To ensure that every PMBC survivor receives reliable information and support regarding favorable lifestyle change, we suggest offering this during each phase of the care continuum.

## Supporting information

S1 File Interview topic list. (PDF)
