# Supplementary material for: Determinants and timing of lifestyle changes in postmenopausal breast cancer survivors: A longitudinal qualitative study
Source: PLoS One. 2025 Nov 24;20(11):e0337089. doi: 10.1371/journal.pone.0337089 (PMC12643267; doi:10.1371/journal.pone.0337089)
Supplement: S1 File — (PDF) [file pone.0337089.s001.pdf]

Appendix A. Interview topic list

Phase 1:

- Thank for hospitality and participation in the study
- Introduction of interviewer
- Emphasize there are no right or wrong answers

Phase 2:

- Introduction to the study
- Emphasize everything will be handled confidential/anonymous
- Emphasize everyone is free to stop at any time
- Ask for informal consent to audiotape the interview

Phase 3:

- How are you?
- How did you experience last year (before/after diagnosis; changes; complaints; positive and negative important moments related to lifestyle/body image/trust in body)?
- Do you have previous experiences with cancer in your family (family or genetic)?

Phase 4:

Based on answers on questionnaire OPTIMUM-study Wave 1 (4-6 months following diagnosis breast cancer)

- How did you experience adherence to a recommendation for lifestyle and bodyweight before diagnosis (exercise, diet, sleep, smoking, alcohol)?
- Did this differ before and after the diagnosis breast cancer?
- Did you experience a need for lifestyle/bodyweight support before or after the diagnosis breast cancer?
- What do you experience as barriers/facilitators for maintaining favorable lifestyle changes?
- Did you attempt to change your lifestyle/bodyweight prior to the diagnoses breast cancer? Why (not)? How?

Based on answers on questionnaire OPTIMUM-study Wave 2 (1 year following diagnosis breast cancer)

- How did you experience adherence to a recommendation for lifestyle and bodyweight (exercise, diet, sleep, smoking, alcohol)?
- What makes it (im)possible to adhere to this guideline?
- What do you need to adhere to this guideline (from who and when)?
- What do you currently experience as barriers/facilitators for maintaining favorable lifestyle changes?
- Did you attempt to change your lifestyle/bodyweight during the last year? Why (not)? How?

Phase 5 (finishing off):

- Did you receive any information related to lifestyle or bodyweight during the past year? Did this lead to change?
- Did you receive any support related to lifestyle or bodyweight, for example from a physical therapist or dietician?

Phase 6:

- Thank you for shared information and experiences
- Continuation of the research project: member-checking and interview over half a year
- Feedback & Questions
